# HIV-1 *pol* Diversity among Female Bar and Hotel Workers in Northern Tanzania

**DOI:** 10.1371/journal.pone.0102258

**Published:** 2014-07-08

**Authors:** Ireen E. Kiwelu, Vladimir Novitsky, Elimsaada Kituma, Lauren Margolin, Jeannie Baca, Rachel Manongi, Noel Sam, John Shao, Mary F. McLane, Saidi H. Kapiga, M. Essex

**Affiliations:** 1 Kilimanjaro Christian Medical Centre and College, Tumaini University, Moshi, Tanzania; 2 Department of Immunology and Infectious Diseases, Harvard School of Public Health, Boston, Massachusetts, United States of America; 3 London School of Hygiene and Tropical Medicine, London, United Kingdom; 4 Kilimanjaro Reproductive Health Program, Moshi, Tanzania; Lady Davis Institute for Medical Research, Canada

## Abstract

A national ART program was launched in Tanzania in October 2004. Due to the existence of multiple HIV-1 subtypes and recombinant viruses co-circulating in Tanzania, it is important to monitor rates of drug resistance. The present study determined the prevalence of HIV-1 drug resistance mutations among ART-naive female bar and hotel workers, a high-risk population for HIV-1 infection in Moshi, Tanzania. A partial HIV-1 *pol* gene was analyzed by single-genome amplification and sequencing in 45 subjects (622 *pol* sequences total; median number of sequences per subject, 13; IQR 5–20) in samples collected in 2005. The prevalence of HIV-1 subtypes A1, C, and D, and inter-subtype recombinant viruses, was 36%, 29%, 9% and 27%, respectively. Thirteen different recombination patterns included D/A1/D, C/A1, A1/C/A1, A1/U/A1, C/U/A1, C/A1, U/D/U, D/A1/D, A1/C, A1/C, A2/C/A2, CRF10_CD/C/CRF10_CD and CRF35_AD/A1/CRF35_AD. CRF35_AD was identified in Tanzania for the first time. All recombinant viruses in this study were unique, suggesting ongoing recombination processes among circulating HIV-1 variants. The prevalence of multiple infections in this population was 16% (n = 7). Primary HIV-1 drug resistance mutations to RT inhibitors were identified in three (7%) subjects (K65R plus Y181C; N60D; and V106M). In some subjects, polymorphisms were observed at the RT positions 41, 69, 75, 98, 101, 179, 190, and 215. Secondary mutations associated with NNRTIs were observed at the RT positions 90 (7%) and 138 (6%). In the protease gene, three subjects (7%) had M46I/L mutations. All subjects in this study had HIV-1 subtype-specific natural polymorphisms at positions 36, 69, 89 and 93 that are associated with drug resistance in HIV-1 subtype B. These results suggested that HIV-1 drug resistance mutations and natural polymorphisms existed in this population before the initiation of the national ART program. With increasing use of ARV, these results highlight the importance of drug resistance monitoring in Tanzania.

## Introduction

Antiretroviral therapy (ART) has resulted in dramatic reduction of morbidity and mortality among HIV-1 infected individuals [1]–[3]. However, the emergence of drug-resistant viral variants and their potential spread remains a legitimate concern with serious implications for the course of the epidemic [4]–[7].

A virologic failure during the course of ART regimen is frequently related to HIV drug resistance, which arises from mutations in the genes that encode the molecular targets for the drugs, i.e., the HIV-1 protease (PR) and reverse transcriptase (RT) *pol* gene products. The HIV-1 RT is highly error-prone due to a lack of proofreading capacity, which often results in numerous polymorphisms. If viral mutations are associated with HIV drug resistance, these viral variants can have selective advantage and avoid drug pressure [8]–[10].

HIV-1 mutations associated with drug resistance are classified as either primary (major) or secondary (minor). Primary mutations are selected under drug pressure, may lead to a several-fold decrease in sensitivity to one or more antiretroviral drugs, and are extremely rare in the absence of treatment [11]. Secondary mutations are defined as having little or no effect on drug susceptibility, but may lead to increased resistance or increased replication capacity in the presence of major mutations [11], [12]. Thus the appearance of a primary mutation in a genome already containing secondary mutations could influence the speed with which highly resistant viruses are selected during ART [13].

As access to ART rapidly increases in resource-limited countries, the prevalence of circulating HIV-1 drug resistant strains is also expected to increase. Acquired HIV-1 drug resistance developed during the course of treatment can spread upon viral transmission to newly infected individuals. The transmitted HIV-1 drug resistance may pose a challenge for therapeutic control of infection, by reducing the efficacy of first-line antiretroviral (ARV) treatment, and impact clinical outcome.

ART was introduced to Tanzania in 1995 with mono and dual regimens available to only a small number of patients due to the high cost of the drugs [14], [15]. Access to ART has increased since the Tanzanian government launched its public-sector ART program free of charge in October 2004 [14], [15].

The current standard first-line ART for HIV-1 infection in Tanzania consists of two nucleoside reverse transcriptase inhibitors (NRTIs), zidovudine (ZDV) or stavudine plus lamivudine (3TC), and one non-nucleoside reverse transcriptase inhibitor (NNRTI), nevirapine (NVP) or efavirenz (EFV). If the patient fails to respond to the first-line regimens, the second-line regimens include abacavir/didanosine (ABC/ddI) in combination with lopinavir or saquinavir boosted with ritonavir (LPV/r or SQVr) [14]–[16]. Protease inhibitors (PIs) have been used rarely in Tanzania, and were not available in the public sector at the time the specimens for this study were collected.

Tanzania is one of the African countries severely affected by the HIV/AIDS epidemic with 5.7% of its 40 million people infected with HIV [17]. The HIV-1 subtypes A1, C, and D, as well as CRF10_CD and unique inter- and intra-subtype recombinant viruses, have been reported in Tanzania [18]–[26].

Recently we found that HIV-1 subtypes A1, C, and D, and inter- and intra-subtype recombinant viruses, were prevalent among female bar and hotel workers in Northern Tanzania [21], [26]. HIV-1 subtypes and recombinants may be associated with various phenotypes such as disease progression [27], transmission patterns [28], as well as different pathways of drug resistant evolution [29]–[32].

HIV-1 subtypes may respond differently to ARV regimens [33]–[35]. Within the HIV-1 group M, it has been reported that isolates of subtype D tend to be less susceptible to ZVD, 3TC, ddI, NVP, and ritonavir [35]. Similarly, it has been reported that some subtype G strains have decreased susceptibility to PIs [36], [37]. In HIV-1 CRF01_AE infection, the RT mutations T69N and V75M were seen more frequently than in HIV-1 subtype B [38].

The evolution of drug-resistant mutations in the non-B HIV-1 epidemic may not necessarily follow the patterns observed in HIV-1B infection [32]. However, limited information is available on non-B HIV-1 subtypes, particularly in regions like Tanzania where multiple HIV-1 subtypes A1, C, and D, as well as a high number of unique inter- and intra-subtype recombinant viruses, co-circulate. It is important to estimate the baseline prevalence of viral polymorphisms that might be associated with HIV-1 drug resistance in regions with multiple HIV-1 subtypes.

The present study estimated the prevalence of HIV-1 drug resistance mutations in *pol* within a high-risk population of HIV-1-infected ART-naïve female bar and hotel workers, by single-genome amplification and sequencing (SGA/S) of specimens collected in 2005.

## Methodology

### Ethics statement

This study was conducted according to the principles expressed in the Declaration of Helsinki, and was approved by the research ethics committees at the Kilimanjaro Christian Medical Centre (KCMC), Tanzania National Institute for Medical Research, and Harvard School of Public Health (HSPH). All study subjects provided written informed consent for participation in the study.

### Study population

The samples for this study were collected from treatment-naïve female bar and hotel workers who were enrolled in a prospective cohort study between December 2004 and March 2007. Descriptions of assessment of HIV-1 status, recruitment of study subjects, characteristics of the cohort, and sampling procedures have been provided elsewhere [21], [39], [40]. All subjects enrolled in this study had similar sexual risk behaviors and are considered one of the high-risk populations for HIV-1 infection in Tanzania [21]. Subjects were followed-up quarterly over one year. At each study visit women were examined, consented, and interviewed about their sexual behavior and HIV-related risk factors, and blood samples were collected for further analysis.

Among 800 subjects enrolled in the study, 139 (17%) were HIV-1 positive by serological testing [21], [39], [40]. A subset of 50 out of 139 HIV-1 positive subjects with at least two samples collected one year apart has been recently characterized [21].

In this study we estimated the prevalence of HIV-1 drug resistance mutations and *pol* diversity. Thus a subset of 50 samples collected at enrollment was genotyped. The median age of subjects at study entry was 30 years (IQR 26–37). None of the study subjects reported previous exposure to ART. Viral load in plasma was quantified [21]. The viral load results are shown in Table S1.

### Single-genome amplification and sequencing (SGA/S)

The isolation of peripheral blood mononuclear cells (PBMCs) from whole blood and genomic DNA have been described previously [21]. A fragment of the HIV-1 *pol* gene of about 1,660 bp encoding the entire PR and part of RT (position 2085–3763; HXB2 numbering) was amplified using a modified SGA/S technique [41], [42] based on the limiting dilutions method [43]. The first-round PCR was conducted with primers IBF1 (5′-AAA TGA TGA CAG CAT GTC AGG GAG -3′; nucleotides 1826–1847; HXB2 numbering) and 3891L (5′-TCC TCT GTC AGT AAC ATA CCC TG-3′; nucleotides 3913–3932; HXB2 numbering). PCR amplification was performed in 20 µl and contained 1 µl of proviral DNA, 1.8 mM FastStart High Fidelity Buffer (Roche), 10 mM deoxynucleotide triphosphate (dNTPs (dATP, dCTP, dGTP and dTTP)) (Roche), 10 pmol of each primer (Integrated DNA Technologies) and 5U FastStart High Fidelity Enzyme (Roche). The second-round PCR reaction was done with primers 2018U (5′-TTG GAA ATG TGG AAA GGA AGG AC-3′; nucleotides 2031–2050; HXB2 numbering) and 3775L (5′-TAC TAG GGG AGG GGT ATT AAC A-3′; nucleotides 3797–3815; HXB2 numbering). The reaction was carried out in a final volume of 25 µl and contained 1 µl of the first-round PCR product diluted 1∶50 and 24 µl of master mix containing 1.8 mM FastStart High Fidelity buffer, 10 mM dNTPs, 10 pmol of each primer and 5U FastStart High Fidelity Enzyme. Thermal cycling conditions for both PCR rounds were as follows: 95°C for 2 minutes, followed by 35 cycles at 95°C for 20 sec, 54°C for 20 sec and 72°C for 2 sec with a final extension step at 72°C for 7 min. Reaction mixtures were stored at 4°C until use.

Amplified products were electrophoretically analyzed by applying 5 µl of second PCR amplification product to 1% agarose gel containing ethidium bromide, and visualized under ultraviolet light. Amplicons were purified by Exo-Sap [44] and directly sequenced on both strands on the ABI 3730 DNA analyzer using BigDye technology.

### Phylogenetic analysis and subtype determination

Generated proviral DNA sequences were assembled and edited using SeqScape V 2.7. The *pol* sequences were aligned together with the HIV-1 subtype reference sequences retrieved from the Los Alamos HIV-1 sequence database [45] using the MUSCLE algorithm [46] in MEGA 5.0 [47]. Minor manual adjustment was done by Bioedit version 7.0 [48]. Maximum likelihood (ML) phylogenetic trees were constructed by PhyML version 3.0.1 [49] and visualized by FigTree v1.3.1 [50]. The approximate likelihood ratio test (aLRT) was used as a statistical test for support of splits [51]. aLRT values ≥0.95 were considered significant and are displayed at the tree nodes. The neighbor-joining (NJ) trees were constructed by MEGA 5.0 using the Kimura-two parameter model with 1000 bootsrap replicates [52]. Bootstrap values ≥80% were considered significant [53]. HIV-1 subtypes were determined based on branching topology, clustering and splits support of the analyzed sequences and their phylogenetic relationships with HIV-1 reference subtype sequences from the Los Alamos HIV-1 sequence database, as described elsewhere [21].

The proviral DNA sequences were analyzed for evidence of APOBEG3G-induced hypermutations using Hypermut tool V2.0 [54]. Thirteen quasispecies from five subjects with a p value of ≤0.05 were considered enriched for mutations consistent with APOBEG3G signatures and were excluded from analysis. The final set included 622 *pol* sequences.

### Screening for inter-subtype recombination and breakpoints identification

All sequences generated in this study were screened for evidence of inter-subtype recombination by Recombination Identification Program (RIP 3.0) [45] and REGA HIV-1 Subtyping Tool-Version 2.0 [55]. The identified recombinant viruses were further analyzed for breakpoints identification using bootscan by SimPlot software v3.5.1 [56] as previously described [21]. The HIV-1 subtype reference sequences were retrieved from the Los Alamos HIV Sequence Database [45]. Identified breakpoints were visually inspected in BioEdit. To confirm the HIV-1 subtypes in the inter-subtype recombinant viruses, nucleotide sequences on both sides of the breakpoint were analyzed independently by re-constructing phylogenetic trees using the splits at the putatively identified breakpoints, as described previously [21].

For the sequences with both recombinants and pure subtypes (multiple infections), we established whether or not the recombinant viruses originated within the infected individuals. The recombinant sequences were split at the putatively identified breakpoints, realigned with the pure subtypes which originated from the same subjects together with the reference sequences, including CRFs if required and examined by neighbor-joining phylogenetic tree analysis.

### Multiplicity of HIV-1 infection

To determine HIV-1 infections with multiple viral variants, the HIV-1 *pol* gene analysis was performed as described recently for HIV-1 *env* gene analysis [21].

### Drug resistance mutation analyses

The HIV-1 *pol* quasispecies were evaluated for HIV-1 drug resistance mutations and for naturally occurring polymorphisms in the PR and RT using the International AIDS Society–USA (IAS-USA) major mutation list [57] and the Stanford University HIV Drug Resistance Database [58].

### Control for cross-contamination

Control of laboratory cross-contamination during specimen collection, processing, amplification, and/or sequencing was performed routinely, as described previously [21].

### Statistical analysis

Descriptive statistics were performed using Sigma Stat v.3.5. The bootstrap and aLTR support values for splits in the inferred phylogenetic trees were computed by MEGA 5.0 and PhyML respectively.

### Accession numbers

Sequences have been assigned GenBank database accession numbers KF530900–KF531521.

## Results

### HIV-1 *pol* subtyping

In this study we targeted the same subjects described in our recent study on diversity of the V1-C5 region of HIV-1 *env* gp 120 [21]. A total of 622 *pol* sequences were generated from 45 subjects [21]. The median number of *pol* sequences per subject was 13 (IQR 5–20). Samples from five subjects (codes 86, 181, 321, 404, 945) could not be amplified.

**Figure 1:**
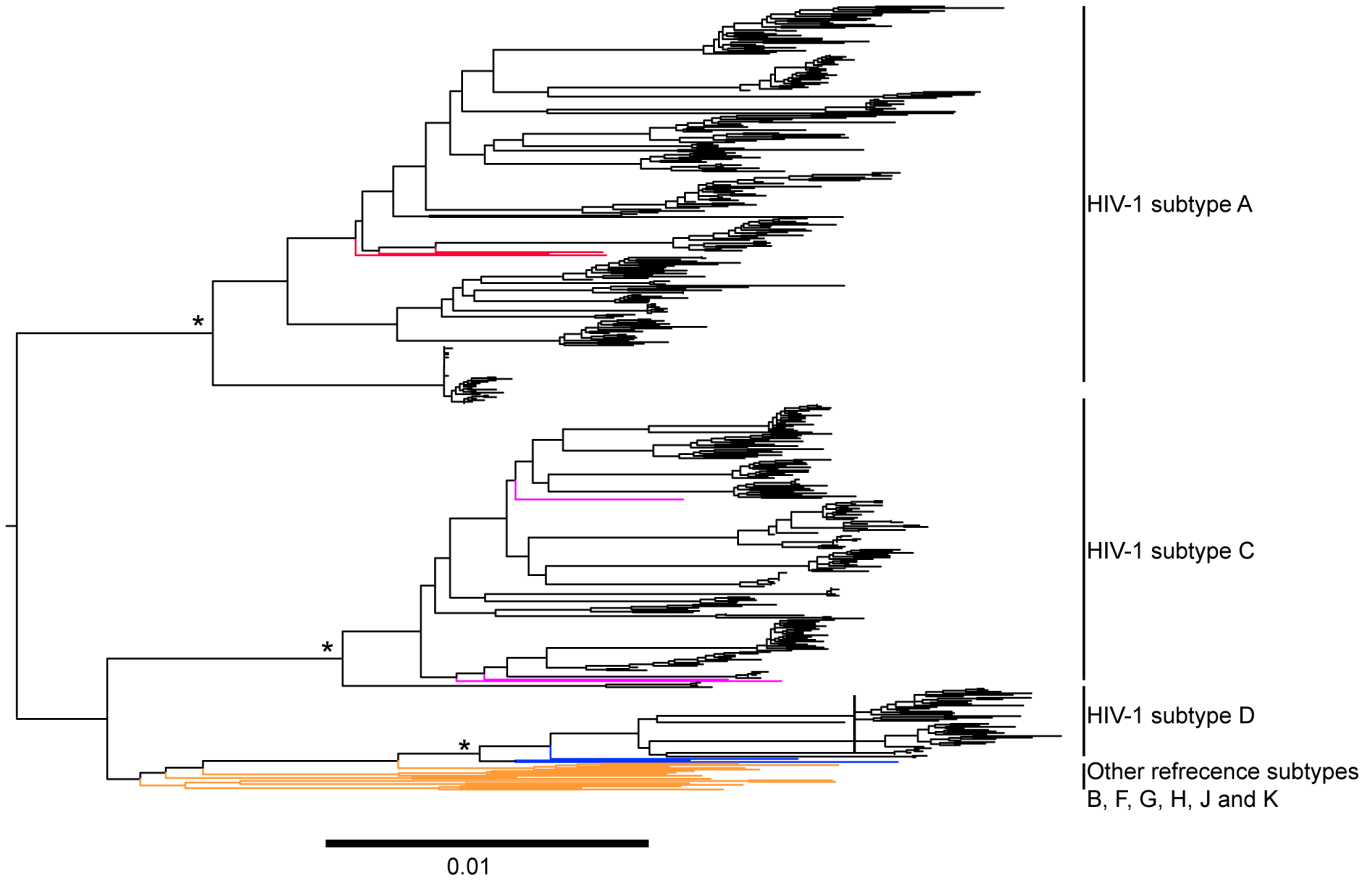
ML phylogenetic tree of HIV-1 protease and reverse transcriptase sequences of the *pol* gene from 45 subjects. The ML was constructed by PhyML 3.0.1 and visualized in FigTree. The tree is rooted with the HIV-1 group N consensus sequence as an outgroup. 488 non-recombinant *pol* sequences generated from the 45 subjects were analyzed with HIV-1 reference subtypes from the Los Alamos HIV-1 Sequence Database. HIV-1 inter-subtype recombinant *pol* DNA sequences were excluded in this figure. HIV-1 reference subtypes A1, C, and D are shown in red, pink and blue, respectively. The other references are shown in orange. Approximate likelihood ratio test (aLRT) values of ≥0.95 were considered significant and are shown by an asterisk (*). The scale at the bottom of the figure corresponds to 0.01 nucleotide substitutions per site.

Analysis of phylogenetic relationships between the generated *pol* sequences revealed that A1 was the most common HIV-1 subtype (35.6%), followed by subtype C (28.9%) and HIV-1 inter-subtype recombinant viruses (26.7%). HIV-1 subtype D was less prevalent (8.9%). Similar results were observed in our previous study on the *V1-C5 env* gene. However, the *pol-*based prevalence of HIV-1 inter-subtype recombinants (26.7%) was higher than the *env-*based prevalence (8.6%) [21], although this finding did not reach statistical significance (p = 0.0513, Fisher exact test). Using the combined *env/pol* data, the overall HIV-1 subtype distribution was A1/A1 (35.6%), C/C (24.4%), D/D (4.4%) and inter-subtype recombinants (35.6%), highlighting a higher rate of HIV-1 inter-subtype recombinants in the combined analysis (Table 1). Figure 1 shows the phylogenetic relationships among a subset of 488 non-recombinant HIV-1 *pol* sequences. The 134 HIV-1 inter-subtype recombinant *pol* DNA sequences were analyzed separately, as their topology in the phylogenetic tree was not informative.

**Table 1:** Distribution of HIV-1 subtypes among female bar and hotel workers in Moshi, Kilimanjaro, Tanzania in 2005.

Subtype	V1-C5 *env* gene*	*pol* gene (PR and RT)	*Env* */*pol* genes combination
A1	24 (53.3%)	16 (35.6%)	16 (35.6%)
C	14 (31.1%)	13 (28.9%)	11 (24.4%)
D	3 (6.7%)	4 (8.9%)	2 (4.4%)
Recombinant	4 (8.9%)	12 (26.7%)	16 (35.6%)
**Total**	**45**	**45**	**45**

*Kiwelu et al., 2013.

### HIV-1 inter-subtype recombinant viruses

The distribution of HIV-1 inter-subtype recombinant viruses in two regions (*env* and *pol*) is shown in Table 2. HIV-1 inter-subtype recombinant viruses were found in 12 (26.7%) of the 45 subjects (Table 1). In seven subjects (codes 33, 87, 355, 558, 733, 838 and 909), all of the quasispecies for the *pol* gene were represented by inter-subtype recombinant viruses, while five subjects (codes 177, 209, 322, 491 and 603) had multiple HIV-1 subtype infections, suggesting possible recombination and/or dual infections in this population. To determine the relationship between the non-recombinant subtypes and the putative recombinant regions based on the *pol* gene, phylogenetic analysis was performed. Results for the *pol* gene showed that in four of the five subjects (codes 177, 322, 491 and 603) with dual infections, the pure subtypes were found to be parental strains of the recombinant viruses, while in the remaining subject (code 209), the pure subtype was not a parental strain of the recombinant virus (data not shown).

**Table 2:** Distribution of HIV-1 V1-C5 *env* and *pol* sequences among female bar and hotel workers in Moshi, Kilimanjaro, Tanzania, with at least one inter-subtype recombinant virus.

Subject code	V1-C5 *env* gene [21]	PR and RT (*pol* gene)
33	D/A1	D/A1/D
87	A1	C/A1
177	A1	A1
		A1/C/A1
209	A1	A1/U*/A1
		C/U*/A1
322	A1	C
	A1/C/A1	C/A1
355	A1	U*/D/U*
471	C/A1	C
491	A1	A1
		C
		D/A1/D
510	D/U*	D
	D/U*/D	
558	C	A1/C
603	C	A1
		A1/C
697	A1	C
740	A1	D
733	D	CRF35_AD/A1/CRF35_AD
838	C	CRF10_CD/C/CRF10_CD
909	A1	A2/C/A2
**Total Recombinants**	**4 (8.6%)**	**12 (26.7%)**

U* unclassified region.

**Figure 2:**
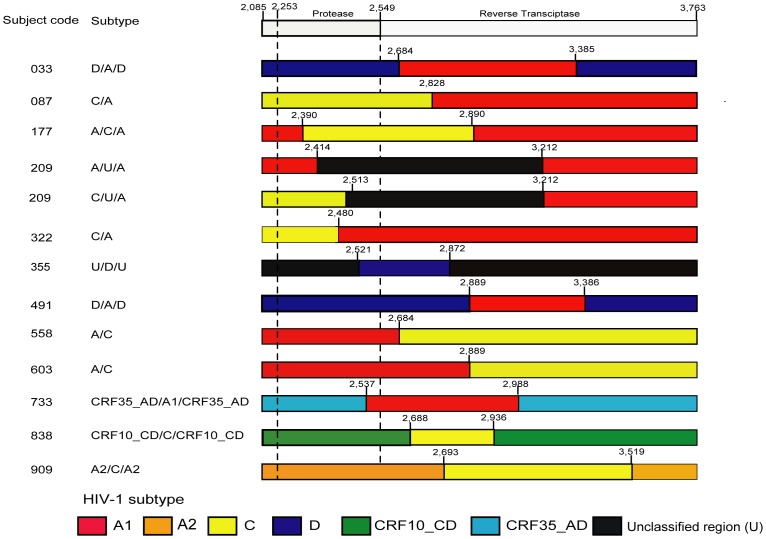
Schematic representation of recombinant viruses detected in 12 subjects showing recombination breakpoints. Localization of breakpoints between HIV-1 subtypes was done by SimPlot analysis and identified breakpoints were visually inspected in BioEdit. The numbers in each bar indicate breakpoints and were given HXB2 numbering. The first bar represents the protease and reverse transcriptase genes. The dashed lines indicate the positions where protease gene starts, 2, 253 (HXB2 numbering), and ends, 2,549 (HXB2 numbering). The genome segments are colored according to HIV-1 subtypes. Red, orange, yellow, blue, green, and light blue represent sequences from HIV-1 subtypes A1, A2, C, D, CRF10_CD and CRF35_AD, respectively. The black bar represents the unclassified (U) regions. Confirmation of recombination was conducted by constructing phylogenetic trees of the putative recombinant regions against reference HIV-1 subtypes.

We also identified two complex circulating recombinant forms (CRFs), CRF10_CD/C/CRF10_CD and CRF35_AD/A1/CRF35_AD in this population (Fig. 2). CRF10_CD has been previously reported in Tanzania [20], [22], [23], while this is the first time that CRF35_AD has been reported in this population as well as in Tanzania. The CRF35_AD/A1/CRF35_AD recombinant was further analyzed to confirm the recombination patterns of the 40 generated viral quasispecies of subject 733 (number of viral quasispecies per subject ranged from 1 to 45 quasispecies). ML trees were generated separately for the three regions, 2,080–2,536, 2,537–2,987, 2988–3746 (HXB2 numbering). Results showed that the first analyzed fragment clustered with HIV-1 CRF35_AD reference (Fig. S1B; aLRT support of 0.88). The second fragment clustered with HIV-1 subtype A1 reference (Fig. S1C; aLRT support of 0.76). The third fragment clustered with HIV-1 CRF35_AD reference (Fig. S1D; aLRT support of 0.93). The low aLRT support value for all the three fragments could possibly be due to the short length and limited number of informative sites. The relationship of these strains to other published HIV-1 *pol* sequences was investigated with the BLAST subtyping tool [59]. The closest available sequence was HIV-1 isolate TV725 from Canada [60]. Similar analyses were performed for the CRF10_CD/C/CRF10_CD recombinant virus to confirm the HIV-1 sub-genomic regions (data not shown).

Phylogenetic analysis of both *env* and *pol* genes indicated 16 (35.6%) of 45 subjects were infected with HIV-1 inter-subtype recombinant viruses. Among these recombinant viruses: two subjects (codes 33 and 322) had recombination breakpoints in both *env* and *pol* regions; ten subjects (codes 87, 177, 209, 355, 491, 558, 603, 733, 838 and 909) had a virus with breakpoints in the *pol* gene only; two subjects (codes 471 and 510) had recombination breakpoints in *env* gene only; and two subjects (codes 697 and 740) had discordant *env* and *pol* subtypes, A1/C and A1/D, respectively.

Recombinant strains were analyzed in detail based on the location of recombination breakpoints. Putative recombinant regions were split according to the breakpoints and analyzed by neighbor-joining trees and HIV-1 reference subtypes. Results for this analysis are shown in Figure 2. Thirteen different recombination patterns were observed in the *pol* gene: 11 recombination patterns were observed in 11 of the 12 subjects with recombinant viruses, while two different recombination patterns were observed in the remaining subject (Fig. 2; code 209). Of note, in the *env* analysis of the same subjects, we observed only five different patterns [26], suggesting that the *pol* region has a high recombination rate in this population.

The HIV-1 inter-subtype recombinant viruses in this study were unique, shared no recombination breakpoints, and demonstrated complex subtype structure. For example, a portion of HIV-1 in subject 209 clustered with none of the group M HIV-1 subtypes. However, when we compared the 18 viral quasispecies of subject 209 with the HIV-1 sequences in GenBank using BLAST [59], the closest available HIV-1 sequences to the 18 viral quasispecies of subject 209 were HIV-1 *pol* sequences from Spain with 94% identity [61].

### HIV-1 multiple infections

The prevalence of multiple HIV-1 infections in this study was 16% (n = 7). Two subjects (codes 66 and 291) were infected with HIV-1 multiple variants of HIV-1 subtype C, while the remaining five subjects (codes 177, 209, 322, 491, and 603) were infected with both pure subtypes and recombinant viruses. Recently we reported that 12 (27%) of 45 subjects had multiple HIV-1 infections based on analysis of the HIV-1 *env* gene [21]. However, congruence between two structural viral genes, *env* and *pol*, in identification of multiplicity of HIV-1 infection was poor, at least in this population. Thus, only one of 12 subjects with multiple *env* infections (code 291) was infected with multiple variants of HIV-1 subtype C based on the *pol* gene analysis. Multiple HIV-1 infection was not confirmed in the other 11 subjects due to non-significant bootstrap support values (6 subjects), low number of quasispecies (2 subjects), or no evidence for multiple distinct variants (3 subjects). At the same time, one subject (code 66) with homogeneous *env* quasispecies indicating HIV-1 infection with a single variant, was classified as infected with multiple HIV-1 variants based on the *pol* gene analysis. A summary of HIV-1 infection with single and multiple viral variants is shown in Table S2.

### Reverse transcriptase inhibitor (RTI) resistance mutations

Table 3 summarizes the mutations and polymorphisms associated with PR and RT inhibitors. Primary HIV-1 drug resistance mutations to RT inhibitors were identified in three (7%; codes 201, 245 and 291) of the 45 subjects. The identified NRTI mutations included D67N and K65R, while the NNRTI mutations were V106M and Y181C. The NRTI-associated polymorphisms were observed at positions 41, 69, 75 and 215. The prevalence of the secondary mutations associated with NNRTI at positions 90 and 138 was 11% (n = 5). Single polymorphisms associated with NNRTIs were detected at positions 98, 101, and 190. A subtype-specific polymorphism at position 179 (V179I) was observed among all 16 (100%) subjects infected with HIV-1 subtype A1. Some subjects harbored multiple secondary mutations (e.g., subject 905 with V90I and E138K) and/or polymorphisms (e.g., subject 237 with A98S and L101Q).The significance of the observed polymorphisms in HIV-1 non-B subtypes is unknown.

**Table 3:** Mutations and polymorphisms at positions associated with drug resistance to PIs, NRTIs, and NNRTIs among female bar and hotel workers in Moshi, Kilimanjaro, Tanzania, in 2005.

Subject code	HIV-1Subtype	Total no. of quasispecies	Mutation position	No. of quasispecies with mutation
			**PI: Primary mutation**	
276	C	10	M46I	1
480	A1	32	M46I	1
733	CRF35_AD/A1/CRF35_AD	40	M46L	1
			**NRTI: Primary mutation**	
245	A1	22	D67N	1
201	C	13	K65R	1
			**NRTI: Polymorphisms**	
740	D	22	M41I	1
66	C	13	T69A	1
245	A1	22	T69P	1
209	A1	24	V75A	1
909	A2/C/A2	21	V75A	1
46	A1	24	T215A	1
			**NNRTI: Primary mutation**	
201	C	13	Y181C	1
491	C	45	V106M	4
			**NNRTI: Secondary mutation**	
168	A1	19	V90I	1
838	CRF10_CD/C/CRF10_DC	16	V90I	2
905	A1	16	V90I	9
			E138K	1
237	A1	13	E138A	5
291	C	32	E138A	2
			**NNRTI: Polymorphisms**	
237	A1	13	A98S	6
			L101Q	1
480	A1	32	G190E	1

### Protease polymorphisms associated with protease inhibitor resistance mutations

**Figure 3:**
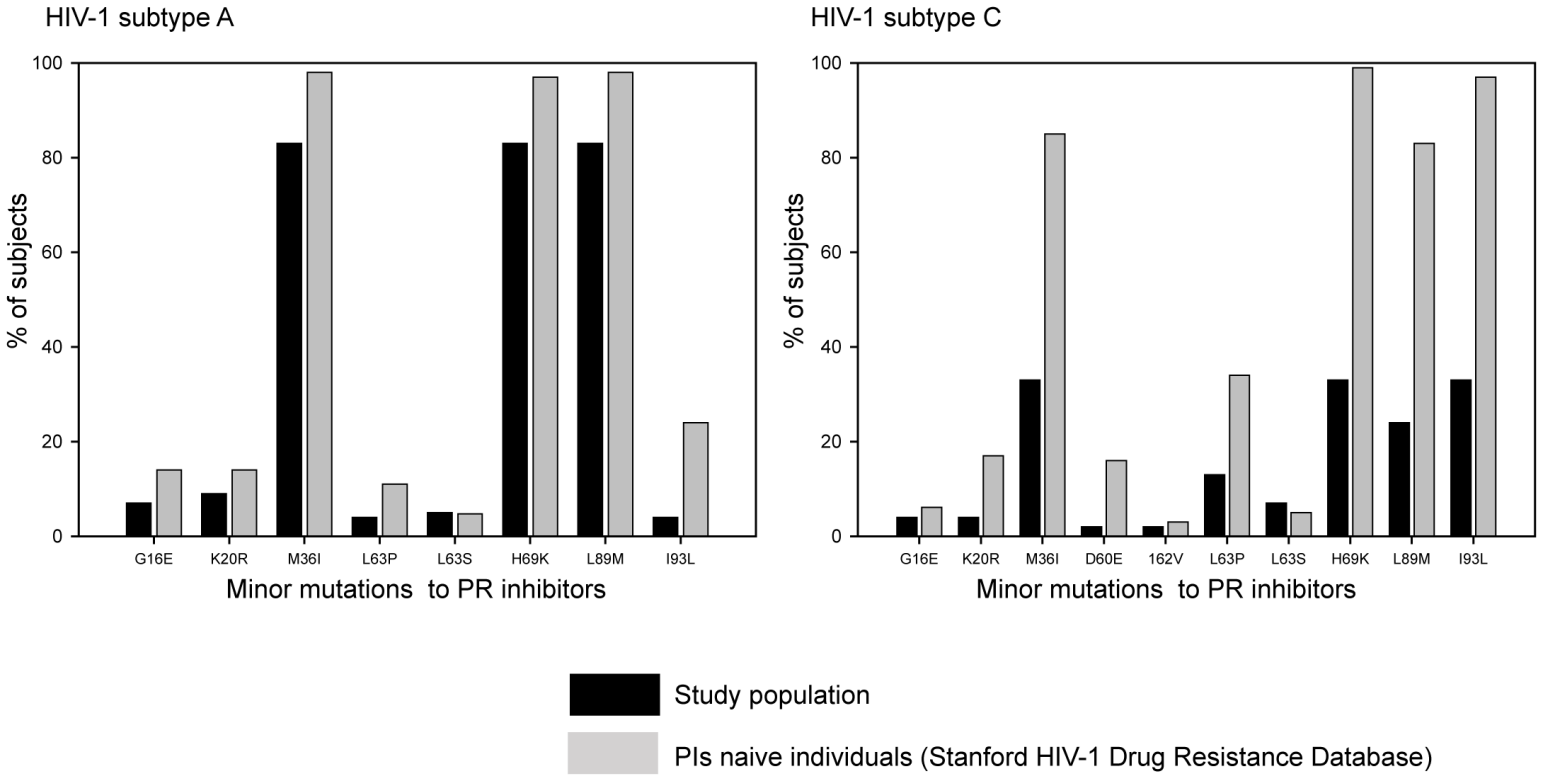
Polymorphisms in protease gene in HIV-1 subtypes A1 and C, compared to ARV-naïve individuals from the Stanford HIV-1 Drug Resistance Database.

In the protease gene, three subjects (7%) had major mutations at position 46 (M46I/L) associated with PR drug resistance mutations (Table 3). Viral polymorphisms were present at multiple positions across protease, e.g., at positions 16, 20, 34, 36, 60, 62, 63, 64, 69, 71, 74, 77, 89, 90 and 93, that are associated with PI-resistance in HIV-1 subtype B. The most frequent polymorphisms were seen at positions M36I (81%), H69K (86%), L89M (74%), and I93L (62%). Given the abundance of viral polymorphisms potentially associated with PI-drug resistance in subtype B, their frequencies were compared with the HIV-1 subtypes A1 and C frequencies in treatment-naïve individuals available from the Stanford HIV Drug Resistance Database (Fig. 3). No statistically significant difference was found (p = 0.161 and p = 0.104, respectively), suggesting HIV-1 subtype-specific polymorphisms are not associated with PI drug resistance.

## Discussion

This study determined the prevalence of HIV-1 subtypes and HIV-1 drug resistance mutations among treatment-naïve female bar and hotel workers, a high-risk population for HIV-1 infection in Moshi, Tanzania. The most prevalent subtype was HIV-1 subtype A1, followed by HIV-1 subtype C, HIV-1 inter-subtype recombinant viruses and HIV-1 subtype D. Similar results were reported in our previous HIV-1 *env-*based study [21]. However, the frequency of HIV-1 inter-subtype recombinant viruses in the HIV-1 *pol* gene (26.7%) showed a trend to be higher (p = 0.051; Fisher exact test) than the frequency observed in the HIV-1 *env* gene (8.6%) in the same population [21]. Similarly, a high prevalence of HIV-1 inter-subtype recombinant viruses was reported in the previous studies using the *pol* gene [62]–[65]. The combined HIV-1 *env* [21] and *pol* prevalence of HIV-1 inter-subtype recombinant viruses was 35.6%. It is possible that near full-length HIV-1 genome analysis could show even higher prevalence of recombinant viruses. Our study supported the previous findings that examining multiple regions of the HIV-1 genome may allow detection of more subjects infected with multiple infections and recombinant viruses [66]–[68]. A high prevalence of HIV-1 inter-subtype recombinant viruses in the HIV-1 *pol* gene suggests that recombination occurs in the *pol* region. These results are consistent with the previous studies demonstrating that *pol* appears to be a hot spot for recombination [62], [63], [66], [69].

The recombination patterns and breakpoints in the HIV-1 *pol* gene were unique in all 12 (26.7%) subjects infected with HIV-1 inter-subtype recombinant viruses. In contrast, in the HIV-1 *env* gene we observed only five recombination patterns in the same population [26]. Additionally, five of the 12 subjects with recombinant viruses had dual infections of pure HIV-1 subtypes and recombinant viruses. The pure HIV-1 subtypes in four subjects were parental strains of the recombinants, suggesting that dual infections were responsible for the generation of these recombinants. Six complex recombinant viruses including circulating recombinant forms (CRFs) were reported in this study: A1/U/A1, C/U/A1, U/D/U, A2/C/A2, CRF10_CD/C/CRF10_CD, and CRF35_AD/A1/CRF35_AD. The complex recombinant virus CRF35_AD/A1/CRF35_AD was not previously reported in Tanzania. The CRF35_AD had been previously described among injecting drug users in Kabul, Afghanistan [60], [70], [71]. The HIV-1 sub-subtype A2 was reported for the first time in Moshi among female bar and hotel workers [72] and was later reported among pregnant women in the Kilimanjaro region [23]. The CRF10_CD recombinant has been previously described in Tanzania [20], [22], [23]. Our results suggest that the HIV-1 sub-subtype A2 and CRF10_CD are present at a low prevalence in this population. The three recombinant variants A1/U/A1, C/U/A1 and U/D/U include regions that did not cluster with any HIV-1 group M subtype, and were therefore considered unclassified regions (U). The source of unclassified regions remains unknown although we cannot exclude a complex recombination between recombinants of unknown degree.

All recombinant viruses identified in this study were unique, and contained the co-circulating HIV-1 subtypes A1, C and D in Tanzania. Similar results were reported in the HIV-1 *env* gene in the same population [21] and in previously published studies in Tanzania [23], [73], [74].

The high prevalence of HIV-1 inter-subtype recombinant viruses in this population may be associated with multiple factors. First, there is the high-risk behavior of women working in hotels and bars in Moshi, Tanzania, who have a high rate of sexual partner change which may facilitate multiple HIV infections and recombinations [73], [75]. Second, co-circulation of HIV-1 subtypes A1, C, D and some other HIV-1 subtypes in this population contributes to the generation of inter-subtype recombinant viruses [21]. Third, analysis of multiple regions of the HIV-1 genome including *env* and *pol* genes allows the detection of more recombinant viruses.

In this study the prevalence of HIV-1 multiple infections was 16% (n = 7 of 45). Two of the seven subjects were infected with multiple HIV-1 variants of the same subtype, while the remaining five subjects were infected with a mixture of pure HIV-1 subtypes and recombinant viruses. However, based on the HIV-1 *env* gene, only 12 (27%) of the 45 subjects were infected with multiple HIV-1 variants of the same subtype [21]. Based on the HIV-1 *pol* gene five more subjects were infected with HIV-1 multiple infections, suggesting that analysis of a single region of the HIV-1 genome may underestimate the true proportion of HIV-1 multiple infections.

Analysis of HIV-1 drug resistance mutations and polymorphisms among female bar and hotel workers revealed that three (7%) of the 45 subjects harbored HIV-1 drug resistance mutations to RT inhibitors, NRTIs and NNRTIs. This is higher than in some previous studies in Tanzania among HIV-1 treatment-naive individuals [15], [64] but is in line with other studies in Tanzania [14], [76]. Our results suggest that HIV-1 strains with drug-resistant mutations to RT inhibitors existed in this population due to suboptimal regimens and adherence during the earlier phase of the HIV/AIDS epidemic in Tanzania, i.e., before the implementation of the national ART program.

Three (7%) subjects had a major mutation at the protease amino acid position 46 (M46I/L) that confers high resistance to protease inhibitors (PIs) only in combination with other mutations, and can occur among untreated persons as natural polymorphisms [77]–[80], as was reported previously in Tanzania among treatment-naïve individuals [14], [15]. Since PIs were not used in Tanzania at the time that the samples were collected, the mutations M46I and M46L most likely represent natural polymorphisms rather than transmitted drug-resistant strains. However, unreported exposure to PI or HIV transmission from individuals receiving PIs cannot be excluded.

All subjects in this study harbored three or more polymorphisms at amino acid positions associated with PIs in HIV-1 subtype B. H69K (86%), M36I (81%), L89M (74%), and I93L (62%) were considered to be subtype-specific natural polymorphisms since they occur at high frequency in HIV-1 subtypes A1, C or D [81]. Data from the Stanford HIV Drug Resistance Database for HIV-1 subtypes A1 and C confirmed that the observed polymorphisms are common among HIV-1 treatment-naïve individuals [58]. Polymorphisms were defined as mutations that occurred in more than 1% of sequences from untreated persons. Subtype-specific polymorphisms were defined as mutations that were significantly more prevalent in each non-B subtype than in subtype B viruses from untreated persons [30]. Subjects with and without HIV-1 drug resistance mutations had similar sexual risk behaviors.

The undisclosed use of ART can be a hidden problem in sub-Saharan Africa. Recently Kahle et al. examined drug levels among subjects with low HIV-1 RNA loads and reported a higher than expected prevalence of unreported ARV drugs use [82]. In this study five subjects were found with primary drug resistant mutations associated with NRTIs, or protease inhibitors. Only one of these subjects, code 201, had plasma viral load below 2.7 log_10_ copies/ml. Due to a shortage of plasma specimens we were not able to measure levels of ART in these subjects, which is a clear study limitation. It would be important to address levels of ART in individuals with drug-resistant mutations and/or low HIV-1 RNA load.

The presence of a high number of substitutions at positions associated with drug resistance mutations in non-B viruses might influence the risk of treatment failure through lowering the genetic barriers to the development of drug resistance [83], [84]. Further studies will be required to gain a better understanding of the clinical and biological implications of the natural polymorphisms at positions associated with drug resistance to PIs and RT inhibitors in non-B HIV-1 subtypes, including the significance of recombinant viruses with the increasing use of ARV drugs.

This study has limitations, some of which have been previously reported [21]. First, analysis of one region of the HIV-1 genome, the *pol* gene (PR and RT), may underestimate the true proportion of HIV-1 subtypes, recombinants and multiplicity of infection. Secondly, the duration and stage of HIV-1 infection were unknown, and the study had no power to determine whether the HIV-1 inter-subtype recombination was due to co-infection, super-infection, or both. Thirdly, in order to detect HIV-1 multiple infections of the same subtype, analysis of multiple viral quasispecies is needed; however, some of the subjects had a relatively low number of quasispecies available. Fourth, some of the subjects had undetectable plasma HIV-1 viral RNA, which is likely to be associated with low efficiency of PCR amplification. In addition, we cannot exclude the possibility that some of the subjects may have been receiving HAART at the time of sample collection without our knowledge.

In conclusion, our study demonstrated that the HIV-1 epidemic in Tanzania is highly diverse, with multiple HIV-1 infections and unique HIV-1 inter-subtype recombinants, as well as complex circulating recombinant forms. HIV-1 subtypes A1 and C are still prevalent in this population, including large proportions of unique HIV-1 inter-subtype recombinant viruses. CRF35_AD was reported for the first time in this population, in Moshi as well as in Tanzania. We have further reported the baseline prevalence of HIV-1 drug resistance mutations and natural polymorphisms at amino acid positions associated with HIV-1 drug resistance to NRTIs, NNRTIs and PIs before ARV drugs were widely used in Tanzania. The results of this study will help to better understand the pathogenesis of HIV-1 infection and the emergence of drug resistance, and should aid in the development of therapeutic strategies in Tanzania.

## Supporting Information

Figure S1 **Maximum likelihood (ML) phylogenetic tress of three segments of the 40 viral quasispecies of subject 733.** Fig. S1A: The bootscan plot generated by SimPlot analysis using a consensus DNA sequence of subject 733 with distinct recombination pattern CRF35_AD/A1/CRF35_AD. HIV-1 subtype A1 is shown in the red bar and CRF35_AD is shown in the blue bar. Fig. S1B is a ML tree of the fragment classified as HIV-1 CRF35_AD, Fig. S1C is a ML tree of the fragment classified as HIV-1 subtype A1, and Fig. S1D is a ML tree of the fragment classified as HIV-1 CRF35_AD. The viral quasispecies of subject 733 are shown in green and the legend at the right of the bootstrap plot indicates reference HIV-1 subtypes. aLRT values ≥0.95 were considered significant and are shown by asterisk (*). Selected aLRT values are shown at the branch node of the tree. Scale at the bottom of the figure corresponds to 0.1 nucleotide substitution per site.
(DOCX)

Table S1 Plasma HIV-1 RNA viral load analyzed from the 45 subjects at the early time point (baseline).
(DOCX)

Table S2 HIV-1 single and multiple variants of the same subtype among female bar and hotel workers in Moshi, Kilimanjaro region, Tanzania.
(DOCX)

## References

[1] Hickman M, Bardsley M, De Angelis D, Ward H (1999) Impact of HIV on adult (15–54) mortality in London: 1979–96. Sex Transm Infect 75: 385–388. 10754940 10.1136/sti.75.6.385 PMC1758260

[2] Palella FJ Jr, Delaney KM, Moorman AC, Loveless MO, Fuhrer J, et al (1998) Declining morbidity and mortality among patients with advanced human immunodeficiency virus infection. HIV Outpatient Study Investigators. N Engl J Med 338: 853–860. 9516219 10.1056/NEJM199803263381301

[3] Whitman S, Murphy J, Cohen M, Sherer R (2000) Marked declines in human immunodeficiency virus-related mortality in Chicago in women, African Americans, Hispanics, young adults, and injection drug users, from 1995 through 1997. Arch Intern Med 160: 365–369. 10668839 10.1001/archinte.160.3.365

[4] Bangsberg DR, Perry S, Charlebois ED, Clark RA, Roberston M, et al (2001) Non-adherence to highly active antiretroviral therapy predicts progression to AIDS. AIDS 15: 1181–1183. 11416722 10.1097/00002030-200106150-00015

[5] Deeks SG (2008) Transmitted minority drug-resistant HIV variants: a new epidemic? PLoS Med 5: e164. 18666826 10.1371/journal.pmed.0050164 PMC2488200

[6] Vandamme AM, Camacho RJ, Ceccherini-Silberstein F, de Luca A, Palmisano L, et al (2011) European recommendations for the clinical use of HIV drug resistance testing: 2011 update. AIDS Rev 13: 77–108. 21587341

[7] Wittkop L, Gunthard HF, de Wolf F, Dunn D, Cozzi-Lepri A, et al (2011) Effect of transmitted drug resistance on virological and immunological response to initial combination antiretroviral therapy for HIV (EuroCoord-CHAIN joint project): a European multicohort study. Lancet Infect Dis 11: 363–371. 21354861 10.1016/S1473-3099(11)70032-9

[8] Ho DD, Neumann AU, Perelson AS, Chen W, Leonard JM, et al (1995) Rapid turnover of plasma virions and CD4 lymphocytes in HIV-1 infection. Nature 373: 123–126. 7816094 10.1038/373123a0

[9] Preston BD, Poiesz BJ, Loeb LA (1988) Fidelity of HIV-1 reverse transcriptase. Science 242: 1168–1171. 2460924 10.1126/science.2460924

[10] Wei X, Ghosh SK, Taylor ME, Johnson VA, Emini EA, et al (1995) Viral dynamics in human immunodeficiency virus type 1 infection. Nature 373: 117–122. 7529365 10.1038/373117a0

[11] Hirsch MS, Conway B, D'Aquila RT, Johnson VA, Brun-Vezinet F, et al (1998) Antiretroviral drug resistance testing in adults with HIV infection: implications for clinical management. International AIDS Society–USA Panel. JAMA 279: 1984–1991. 9643863 10.1001/jama.279.24.1984

[12] Erickson JW, Gulnik SV, Markowitz M (1999) Protease inhibitors: resistance, cross-resistance, fitness and the choice of initial and salvage therapies. AIDS 13 Suppl A: S189–204. 10885776

[13] Vergne L, Peeters M, Mpoudi-Ngole E, Bourgeois A, Liegeois F, et al (2000) Genetic diversity of protease and reverse transcriptase sequences in non-subtype-B human immunodeficiency virus type 1 strains: evidence of many minor drug resistance mutations in treatment-naive patients. J Clin Microbiol 38: 3919–3925. 11060045 10.1128/jcm.38.11.3919-3925.2000 PMC87518

[14] Kasang C, Kalluvya S, Majinge C, Stich A, Bodem J, et al (2011) HIV drug resistance (HIVDR) in antiretroviral therapy-naive patients in Tanzania not eligible for WHO threshold HIVDR survey is dramatically high. PLoS One 6: e23091. 21886779 10.1371/journal.pone.0023091 PMC3158766

[15] Somi GR, Kibuka T, Diallo K, Tuhuma T, Bennett DE, et al (2008) Surveillance of transmitted HIV drug resistance among women attending antenatal clinics in Dar es Salaam, Tanzania. Antivir Ther 13 Suppl 2: 77–82. 18575194

[16] National AIDS Control Programme (2007) HIV/AIDS/STI Surveillance Report, January–December 2005. Dar es Salaam, Tanzania: United Republic of Tanzania Ministry of Health. Report Number 20.

[17] Tanzania Commission for AIDS (TACAIDS), Zanzibar AIDS Commission (ZAC), National Bureau of Statistics (NBS), Office of the Chief Government Statistician (OCGS), Macro International Inc. (2008) Tanzania HIV/AIDS and Malaria Indicator Survey 2007–08. Dar es Salaam, Tanzania: TACAIDS, ZAC, NBS, OCGS, and Macro International Inc.

[18] Arroyo MA, Hoelscher M, Sanders-Buell E, Herbinger KH, Samky E, et al (2004) HIV type 1 subtypes among blood donors in the Mbeya region of southwest Tanzania. AIDS Res Hum Retroviruses 20: 895–901. 15320994 10.1089/0889222041725235

[19] Holm-Hansen C, Ayehunie S, Johansson B, Nkya W, Shao J, et al (1996) HIV-1 proviral DNA sequences of env gp41 PCR amplificates from Tanzania. APMIS 104: 459–464. 8774676

[20] Kiwelu IE, Koulinska IN, Nkya WM, Shao J, Kapiga S, et al (2005) Identification of CRF10_CD viruses among bar and hotel workers in Moshi, Northern Tanzania. AIDS Res Hum Retroviruses 21: 897–900. 16225419 10.1089/aid.2005.21.897

[21] Kiwelu IE, Novitsky V, Margolin L, Baca J, Manongi R, et al (2012) HIV-1 subtypes and recombinants in Northern Tanzania: distribution of viral quasispecies. PLoS One 7: e47605. 23118882 10.1371/journal.pone.0047605 PMC3485255

[22] Koulinska IN, Ndung'u T, Mwakagile D, Msamanga G, Kagoma C, et al (2001) A new human immunodeficiency virus type 1 circulating recombinant form from Tanzania. AIDS Res Hum Retroviruses 17: 423–431. 11282011 10.1089/088922201750102508

[23] Nyombi BM, Kristiansen KI, Bjune G, Muller F, Holm-Hansen C (2008) Diversity of human immunodeficiency virus type 1 subtypes in Kagera and Kilimanjaro regions, Tanzania. AIDS Res Hum Retroviruses 24: 761–769. 18507522 10.1089/aid.2007.0311

[24] Renjifo B, Chaplin B, Mwakagile D, Shah P, Vannberg F, et al (1998) Epidemic expansion of HIV type 1 subtype C and recombinant genotypes in Tanzania. AIDS Res Hum Retroviruses 14: 635–638. 9591718 10.1089/aid.1998.14.635

[25] Siwka W, Schwinn A, Baczko K, Pardowitz I, Mhalu F, et al (1994) vpu and env sequence variability of HIV-1 isolates from Tanzania. AIDS Res Hum Retroviruses 10: 1753–1754. 7888237 10.1089/aid.1994.10.1753

[26] Kiwelu IE, Novitsky V, Margolin L, Baca J, Manongi R, et al (2013) Frequent Intra-Subtype Recombination among HIV-1 Circulating in Tanzania. PLoS One 8: e71131. 23940702 10.1371/journal.pone.0071131 PMC3733632

[27] Kanki PJ, Hamel DJ, Sankale JL, Hsieh C, Thior I, et al (1999) Human immunodeficiency virus type 1 subtypes differ in disease progression. J Infect Dis 179: 68–73. 9841824 10.1086/314557

[28] Chan DJ, Begley K, Smith DE (2009) HIV-1 transmission amongst men who have sex with men: a probabilistic model incorporating antiretroviral treatment optimism-scepticism, sexual beliefs and sexual behaviour. Curr HIV Res 7: 231–236. 19275592 10.2174/157016209787581463

[29] Gu Z, Gao Q, Faust EA, Wainberg MA (1995) Possible involvement of cell fusion and viral recombination in generation of human immunodeficiency virus variants that display dual resistance to AZT and 3TC. J Gen Virol 76 Pt 10: 2601–2605. 7595365 10.1099/0022-1317-76-10-2601

[30] Kantor R, Katzenstein DA, Efron B, Carvalho AP, Wynhoven B, et al (2005) Impact of HIV-1 subtype and antiretroviral therapy on protease and reverse transcriptase genotype: results of a global collaboration. PLoS Med 2: e112. 15839752 10.1371/journal.pmed.0020112 PMC1087220

[31] Martinez-Picado J, Savara AV, Shi L, Sutton L, D'Aquila RT (2000) Fitness of human immunodeficiency virus type 1 protease inhibitor-selected single mutants. Virology 275: 318–322. 10998332 10.1006/viro.2000.0527

[32] Novitsky V, Wester CW, DeGruttola V, Bussmann H, Gaseitsiwe S, et al (2007) The reverse transcriptase 67N 70R 215Y genotype is the predominant TAM pathway associated with virologic failure among HIV type 1C-infected adults treated with ZDV/ddI-containing HAART in southern Africa. AIDS Res Hum Retroviruses 23: 868–878. 17678469 10.1089/aid.2006.0298

[33] Brenner B, Turner D, Oliveira M, Moisi D, Detorio M, et al (2003) A V106M mutation in HIV-1 clade C viruses exposed to efavirenz confers cross-resistance to non-nucleoside reverse transcriptase inhibitors. AIDS 17: F1–5. 12478089 10.1097/00002030-200301030-00001

[34] Grossman Z, Vardinon N, Chemtob D, Alkan ML, Bentwich Z, et al (2001) Genotypic variation of HIV-1 reverse transcriptase and protease: comparative analysis of clade C and clade B. AIDS 15: 1453–1460. 11504976 10.1097/00002030-200108170-00001

[35] Palmer S, Alaeus A, Albert J, Cox S (1998) Drug susceptibility of subtypes A,B,C,D, and E human immunodeficiency virus type 1 primary isolates. AIDS Res Hum Retroviruses 14: 157–162. 9462926 10.1089/aid.1998.14.157

[36] Apetrei C, Descamps D, Collin G, Loussert-Ajaka I, Damond F, et al (1998) Human immunodeficiency virus type 1 subtype F reverse transcriptase sequence and drug susceptibility. J Virol 72: 3534–3538. 9557632 10.1128/jvi.72.5.3534-3538.1998 PMC109572

[37] Descamps D, Apetrei C, Collin G, Damond F, Simon F, et al (1998) Naturally occurring decreased susceptibility of HIV-1 subtype G to protease inhibitors. AIDS 12: 1109–1111. 9662212

[38] Brindeiro R, Vanderborght B, Caride E, Correa L, Oravec RM, et al (1999) Sequence diversity of the reverse transcriptase of human immunodeficiency virus type 1 from untreated Brazilian individuals. Antimicrob Agents Chemother 43: 1674–1680. 10390221 10.1128/aac.43.7.1674 PMC89342

[39] Ao TT, Sam N, Kiwelu I, Mahal A, Subramanian SV, et al (2011) Risk factors of alcohol problem drinking among female bar/hotel workers in Moshi, Tanzania: a multi-level analysis. AIDS Behav 15: 330–339. 21082340 10.1007/s10461-010-9849-y

[40] Ao TT, Sam NE, Masenga EJ, Seage GR 3rd, Kapiga SH (2006) Human immunodeficiency virus type 1 among bar and hotel workers in northern Tanzania: the role of alcohol, sexual behavior, and herpes simplex virus type 2. Sex Transm Dis 33: 163–169. 16505740 10.1097/01.olq.0000187204.57006.b3

[41] Palmer S, Kearney M, Maldarelli F, Halvas EK, Bixby CJ, et al (2005) Multiple, linked human immunodeficiency virus type 1 drug resistance mutations in treatment-experienced patients are missed by standard genotype analysis. J Clin Microbiol 43: 406–413. 15635002 10.1128/JCM.43.1.406-413.2005 PMC540111

[42] Salazar-Gonzalez JF, Bailes E, Pham KT, Salazar MG, Guffey MB, et al (2008) Deciphering human immunodeficiency virus type 1 transmission and early envelope diversification by single-genome amplification and sequencing. J Virol 82: 3952–3970. 18256145 10.1128/JVI.02660-07 PMC2293010

[43] Liu SL, Rodrigo AG, Shankarappa R, Learn GH, Hsu L, et al (1996) HIV quasispecies and resampling. Science 273: 415–416. 8677432 10.1126/science.273.5274.415

[44] Dugan KA, Lawrence HS, Hares DR, Fisher CL, Budowle B (2002) An improved method for post-PCR purification for mtDNA sequence analysis. J Forensic Sci 47: 811–818. 12136989

[45] HIV Sequence Database. Los Alamos National Laboratory. Available: http://www.hiv.lanl.gov.

[46] Edgar RC (2004) MUSCLE: multiple sequence alignment with high accuracy and high throughput. Nucleic Acids Res 32: 1792–1797. 15034147 10.1093/nar/gkh340 PMC390337

[47] Tamura K, Dudley J, Nei M, Kumar S (2007) MEGA4: Molecular Evolutionary Genetics Analysis (MEGA) software version 4.0. Mol Biol Evol 24: 1596–1599. 17488738 10.1093/molbev/msm092

[48] Hall TA (1999) BioEdit: a user-friendly biological sequence alignment editor and analysis program for Windows 95/98/NT. Nucl Acids Symp Ser 41: 95–98.

[49] Guindon S, Gascuel O (2003) A simple, fast, and accurate algorithm to estimate large phylogenies by maximum likelihood. Syst Biol 52: 696–704. 14530136 10.1080/10635150390235520

[50] Rambaut A (2009) FigTree. v1.3.1. Available: http://tree.bio.ed.ac.uk/software/.

[51] Anisimova M, Gascuel O (2006) Approximate likelihood-ratio test for branches: A fast, accurate, and powerful alternative. Syst Biol 55: 539–552. 16785212 10.1080/10635150600755453

[52] Kumar S, Nei M, Dudley J, Tamura K (2008) MEGA: a biologist-centric software for evolutionary analysis of DNA and protein sequences. Brief Bioinform 9: 299–306. 18417537 10.1093/bib/bbn017 PMC2562624

[53] Hills DBJ (1993) An empirical test of bootstrapping as a method for assessing confidence in phylogenetic trees. Syst Biol 42: 182–192.

[54] Rose PP, Korber BT (2000) Detecting hypermutations in viral sequences with an emphasis on G→A hypermutation. Bioinformatics 16: 400–401. 10869039 10.1093/bioinformatics/16.4.400

[55] de Oliveira T, Deforche K, Cassol S, Rambaut A, Vandamme A-M (2006) REGA HIV-1 Subtyping Tool - Version 2.0. Stanford University HIV Drug Resistance Database. Available from http://dbpartners.stanford.edu/RegaSubtyping/.

[56] Lole KS, Bollinger RC, Paranjape RS, Gadkari D, Kulkarni SS, et al (1999) Full-length human immunodeficiency virus type 1 genomes from subtype C-infected seroconverters in India, with evidence of intersubtype recombination. J Virol 73: 152–160. 9847317 10.1128/jvi.73.1.152-160.1999 PMC103818

[57] Johnson VA, Calvez V, Gunthard HF, Paredes R, Pillay D, et al (2011) 2011 update of the drug resistance mutations in HIV-1. Top Antivir Med 19: 156–164. 22156218 PMC6148877

[58] HIV Drug Resistance Database. Stanford University. Available: http://hivdb.stanford.edu/.

[59] Nucleotide Blast. BLAST: Basic Logal Alignment Search Tool. National Library of Medicine. Available: http://blast.ncbi.nlm.nih.gov/Blast.cgi.

[60] Quesnel-Vallieres M, Kouzayha I, Tran E, Barry I, Lasgi C, et al (2011) Novel HIV-1 recombinant forms in antenatal cohort, Montreal, Quebec, Canada. Emerg Infect Dis 17: 271–274. 21291604 10.3201/eid1702.100629 PMC3204757

[61] Pernas M, Casado C, Fuentes R, Perez-Elias MJ, Lopez-Galindez C (2006) A dual superinfection and recombination within HIV-1 subtype B 12 years after primoinfection. J Acquir Immune Defic Syndr 42: 12–18. 16763489 10.1097/01.qai.0000214810.65292.73

[62] Eshleman SH, Gonzales MJ, Becker-Pergola G, Cunningham SC, Guay LA, et al (2002) Identification of Ugandan HIV type 1 variants with unique patterns of recombination in pol involving subtypes A and D. AIDS Res Hum Retroviruses 18: 507–511. 12015904 10.1089/088922202317406655 PMC2573392

[63] Hue S, Hassan AS, Nabwera H, Sanders EJ, Pillay D, et al (2012) HIV type 1 in a rural coastal town in Kenya shows multiple introductions with many subtypes and much recombination. AIDS Res Hum Retroviruses 28: 220–224. 21770741 10.1089/aid.2011.0048 PMC3275924

[64] Nyombi BM, Holm-Hansen C, Kristiansen KI, Bjune G, Muller F (2008) Prevalence of reverse transcriptase and protease mutations associated with antiretroviral drug resistance among drug-naive HIV-1 infected pregnant women in Kagera and Kilimanjaro regions, Tanzania. AIDS Res Ther 5: 13. 18570675 10.1186/1742-6405-5-13 PMC2443165

[65] Rusine J, Jurriaans S, van de Wijgert J, Cornelissen M, Kateera B, et al (2012) Molecular and phylogeographic analysis of human immuno-deficiency virus type 1 strains infecting treatment-naive patients from Kigali, Rwanda. PLoS One 7: e42557. 22905148 10.1371/journal.pone.0042557 PMC3419187

[66] Jetzt AE, Yu H, Klarmann GJ, Ron Y, Preston BD, et al (2000) High rate of recombination throughout the human immunodeficiency virus type 1 genome. J Virol 74: 1234–1240. 10627533 10.1128/jvi.74.3.1234-1240.2000 PMC111457

[67] Piantadosi A, Ngayo MO, Chohan B, Overbaugh J (2008) Examination of a second region of the HIV type 1 genome reveals additional cases of superinfection. AIDS Res Hum Retroviruses 24: 1221. 18729772 10.1089/aid.2008.0100 PMC2743231

[68] Ssemwanga D, Lyagoba F, Ndembi N, Mayanja BN, Larke N, et al (2011) Multiple HIV-1 infections with evidence of recombination in heterosexual partnerships in a low risk Rural Clinical Cohort in Uganda. Virology 411: 113–131. 21239033 10.1016/j.virol.2010.12.025 PMC3041926

[69] Becker-Pergola G, Kataaha P, Johnston-Dow L, Fung S, Jackson JB, et al (2000) Analysis of HIV type 1 protease and reverse transcriptase in antiretroviral drug-naive Ugandan adults. AIDS Res Hum Retroviruses 16: 807–813. 10826487 10.1089/088922200308800

[70] Sanders-Buell E, Saad MD, Abed AM, Bose M, Todd CS, et al (2007) A nascent HIV type 1 epidemic among injecting drug users in Kabul, Afghanistan is dominated by complex AD recombinant strain, CRF35_AD. AIDS Res Hum Retroviruses 23: 834–839. 17604548 10.1089/aid.2006.0299

[71] Soheilli ZS, Ataiee Z, Tootian S, Zadsar M, Amini S, et al (2009) Presence of HIV-1 CRF35_AD in Iran. AIDS Res Hum Retroviruses 25: 123–124. 19182925 10.1089/aid.2008.0199

[72] Kiwelu IE, Renjifo B, Chaplin B, Sam N, Nkya WM, et al (2003) HIV type 1 subtypes among bar and hotel workers in Moshi, Tanzania. AIDS Res Hum Retroviruses 19: 57–64. 12596722 10.1089/08892220360473970

[73] Herbinger KH, Gerhardt M, Piyasirisilp S, Mloka D, Arroyo MA, et al (2006) Frequency of HIV type 1 dual infection and HIV diversity: analysis of low- and high-risk populations in Mbeya Region, Tanzania. AIDS Res Hum Retroviruses 22: 599–606. 16831083 10.1089/aid.2006.22.599

[74] Hoelscher M, Dowling WE, Sanders-Buell E, Carr JK, Harris ME, et al (2002) Detection of HIV-1 subtypes, recombinants, and dual infections in east Africa by a multi-region hybridization assay. AIDS 16: 2055–2064. 12370505 10.1097/00002030-200210180-00011

[75] Kapiga SH, Sam NE, Shao JF, Renjifo B, Masenga EJ, et al (2002) HIV-1 epidemic among female bar and hotel workers in northern Tanzania: risk factors and opportunities for prevention. J Acquir Immune Defic Syndr 29: 409–417. 11917247 10.1097/00126334-200204010-00013

[76] Mosha F, Urassa W, Aboud S, Lyamuya E, Sandstrom E, et al (2011) Prevalence of genotypic resistance to antiretroviral drugs in treatment-naive youths infected with diverse HIV type 1 subtypes and recombinant forms in Dar es Salaam, Tanzania. AIDS Res Hum Retroviruses 27: 377–382. 20954839 10.1089/aid.2010.0113

[77] Bennett DE, Camacho RJ, Otelea D, Kuritzkes DR, Fleury H, et al (2009) Drug resistance mutations for surveillance of transmitted HIV-1 drug-resistance: 2009 update. PLoS One 4: e4724. 19266092 10.1371/journal.pone.0004724 PMC2648874

[78] Birk M, Sonnerborg A (1998) Variations in HIV-1 pol gene associated with reduced sensitivity to antiretroviral drugs in treatment-naive patients. AIDS 12: 2369–2375. 9875574 10.1097/00002030-199818000-00005

[79] Shafer RW, Schapiro JM (2008) HIV-1 drug resistance mutations: an updated framework for the second decade of HAART. AIDS Rev 10: 67–84. 18615118 PMC2547476

[80] Shekelle P, Maglione M, Geotz MB, Wagner G, Wang Z, et al (2007) Antiretroviral (ARV) drug resistance in the developing world. Evid Rep Technol Assess (Full Rep) 1–74. PMC4781335 18088163

[81] Kantor R, Katzenstein D (2004) Drug resistance in non-subtype B HIV-1. J Clin Virol 29: 152–159. 14962783 10.1016/S1386-6532(03)00115-X

[82] Kahle EM, Kashuba A, Baeten JM, Fife KH, Celum C, et al (2014) Unreported antiretroviral use by HIV-1-infected participants enrolling in a prospective research study. J Acquir Immune Defic Syndr 65: e90–94. 24442233 10.1097/QAI.0b013e3182a2db02 PMC3898592

[83] Kijak GH, Currier JR, Tovanabutra S, Cox JH, Michael NL, et al (2004) Lost in translation: implications of HIV-1 codon usage for immune escape and drug resistance. AIDS Rev 6: 54–60. 15168741

[84] Perno CF, Cozzi-Lepri A, Balotta C, Forbici F, Violin M, et al (2001) Secondary mutations in the protease region of human immunodeficiency virus and virologic failure in drug-naive patients treated with protease inhibitor-based therapy. J Infect Dis 184: 983–991. 11574912 10.1086/323604

