# Supplementary material for: HIV-1 pol Diversity among Female Bar and Hotel Workers in Northern Tanzania
Source: PLoS One. 2014 Jul 8;9(7):e102258. doi: 10.1371/journal.pone.0102258 (PMC4087014; doi:10.1371/journal.pone.0102258)
Supplement: Figure S1 — Maximum likelihood (ML) phylogenetic tress of three segments of the 40 viral quasispecies of subject 733. Fig. S1A: The bootscan plot generated by SimPlot analysis using a consensus DNA sequence of subject 733 with distinct recombination pattern CRF35_AD/A1/CRF35_AD. HIV-1 subtype A1 is shown in the red bar and CRF35_AD is shown in the blue bar. Fig. S1B is a ML tree of the fragment classified as HIV-1 CRF35_AD, Fig. S1C is a ML tree of the fragment classified as HIV-1 subtype A1, and Fig. S1D is a ML tree of the fragment classified as HIV-1 CRF35_AD. The viral quasispecies of subject 733 are shown in green and the legend at the right of the bootstrap plot indicates reference HIV-1 subtypes. aLRT values ≥0.95 were considered significant and are shown by asterisk (*). Selected aLRT values are shown at the branch node of the tree. Scale at the bottom of the figure corresponds to 0.1 nucleotide substitution per site. (DOCX) [file pone.0102258.s001.docx]

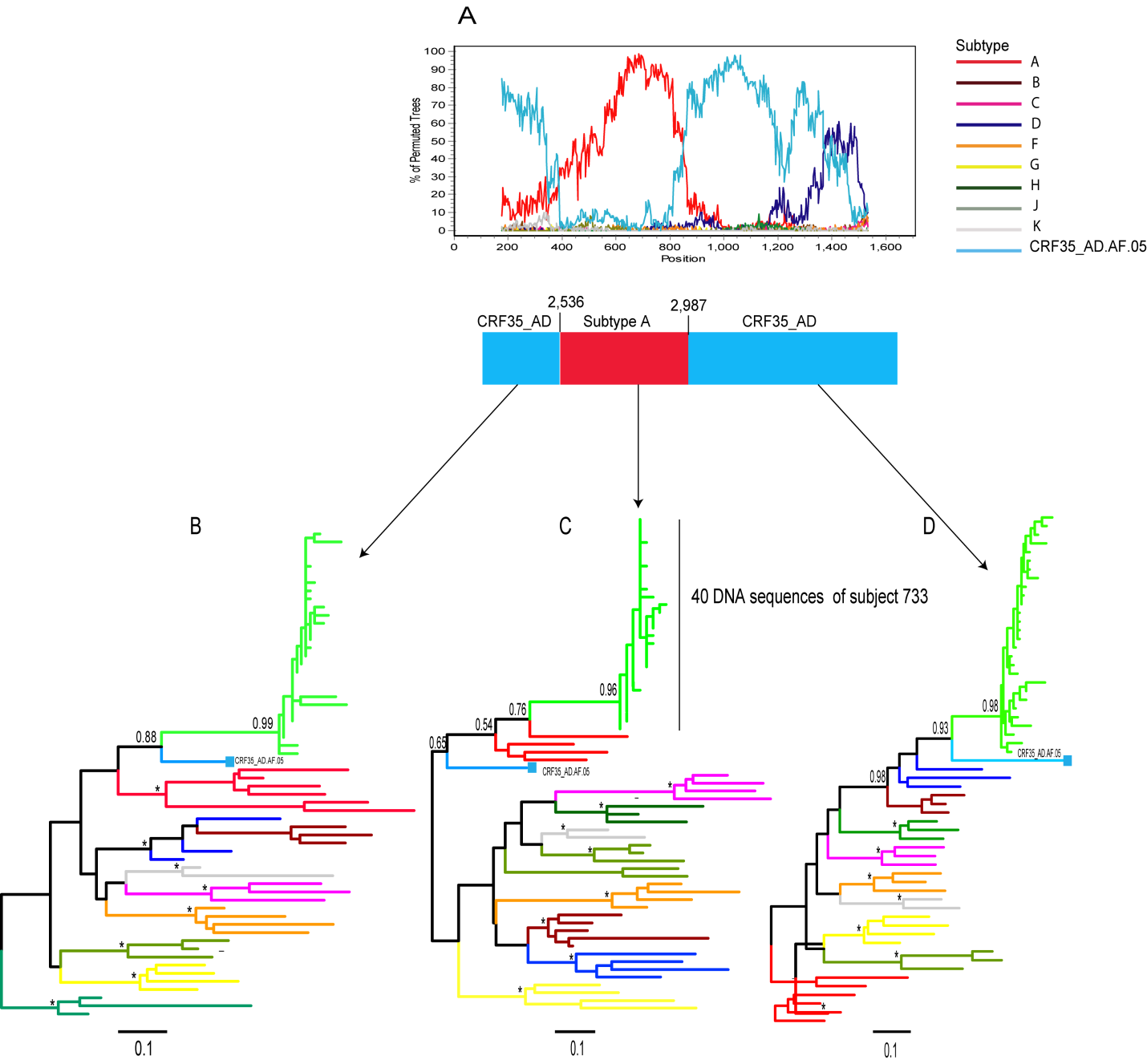


**Figure S1: Maximum likelihood (ML) phylogenetic tress of three segments of the 40 viral quasispecies of subject 733. Fig. S1A:** The bootscan plot generated by SimPlot analysis using a consensus DNA sequence of subject 733 with distinct recombination pattern, CRF35_AD/A/CRF35_AD. HIV-1 subtype A1 is shown in the red bar and CRF35_AD is shown in the blue bar. **Fig. S1B** is a ML tree of the fragment classified as HIV-1 CRF35_AD, **Fig. S1C** is a ML tree of the fragment classified as HIV-1 subtype A1 and **Fig. S1D** is a ML tree of the fragment classified as HIV-1 CRF35_AD. The viral quasispecies of subject 733 are shown in green and the legend at the right of the bootstrap plot indicates reference HIV-1 subtypes. The aLRT values ≥0.95 were considered significant and are shown by asterisk (*). Selected aLRT values are shown at the branch node of the tree. Scale at the bottom of the figure corresponds to 0.1 nucleotide substitution per site.
