# Supplementary material for: HIV-1 pol Diversity among Female Bar and Hotel Workers in Northern Tanzania
Source: PLoS One. 2014 Jul 8;9(7):e102258. doi: 10.1371/journal.pone.0102258 (PMC4087014; doi:10.1371/journal.pone.0102258)
Supplement: Table S1 — Plasma HIV-1 RNA viral load analyzed from the 45 subjects at the early time point (baseline). (DOCX) [file pone.0102258.s002.docx]

**Table S1: Plasma HIV-1 RNA viral load analyzed from the 45 subjects at the early time point (baseline).**

| **Subject code** | **Age** | ***env* HIV-1Subtype** | ***pol* HIV-1 subtype** | **Plasma HIV-1 RNA load Log_10_ copies/ml (baseline visit )** |
| --- | --- | --- | --- | --- |
| 7 | 50 | A1 | A1 | 3.7 |
| 107 | 34 | A1 | A1 | 5.3 |
| 178 | 53 | A1 | A1 | 4.0 |
| 190 | 40 | A1 | A1 | 2.3 |
| 480 | 36 | A1 | A1 | 5.5 |
| 405 | 29 | A1 | A1 | 3.2 |
| 794 | 23 | A1 | A1 | 4.8 |
| 807 | 22 | A1 | A1 | 4.4 |
| 905 | 45 | A1 | A1 | 4.5 |
| 20 | 21 | A1 | A1 | 5.1 |
| 46 | 21 | A1 | A1 | 5.1 |
| 65 | 32 | A1 | A1 | 4.9 |
| 168 | 30 | A1 | A1 | 5.6 |
| 237 | 40 | A1 | A1 | 5.5 |
| 245 | 29 | A1 | A1 | 5.4 |
| 620 | 35 | A1 | A1 | 5.1 |
| 27 | 41 | C | C | 6.0 |
| 63 | 35 | C | C | 5.3 |
| 66 | 36 | C | C | 4.2 |
| 80 | 28 | C | C | 3.2 |
| 276 | 22 | C | C | 4.5 |
| 171 | 38 | C | C | 5.0 |
| 201 | 24 | C | C | 2.7 |
| 291 | 22 | C | C | 5.1 |
| 497 | 22 | C | C | 4.8 |
| 498 | 28 | C | C | 4.9 |
| 968 | 38 | C | C | 5.4 |
| 530 | 23 | D | D | 4.0 |
| 871 | 23 | D | D | 2.5 |
| 33 | 30 | D/A1 | D/A1/D | 3.5 |
| 87 | 29 | A1 | C/A1 | 4.3 |
| 177 | 28 | A1 | A1, A1/C/A1 | 4.9 |
| 209 | 22 | A1 | A1, A1/U*/A1, C/U*/A1 | 4.1 |
| 322 | 31 | A1, A1/C/A1 | C, C/A1 | 4.1 |
| 355 | 25 | A1 | U*/D/U* | 4.2 |
| 471 | 47 | C/A1 | C | 5.6 |
| 491 | 37 | A1 | A1, C, D/A1/D | 4.6 |
| 510 | 23 | D/U*, D/U*/D | D | 4.3 |
| 558 | 35 | C | A1/C | 5.5 |
| 603 | 46 | C | A1, A1/C | 5.0 |
| 697 | 30 | A1 | C | 4.0 |
| 740 | 30 | A1 | D | 6.3 |
| 733 | 37 | D | CRF35_AD/A1/CRF35_AD | 5.3 |
| 838 | 26 | C | CRF10_CD/C/CRF10_CD | 4.2 |
| 909 | 26 | A1 | A2/C/A2 | 5.7 |

U* unclassified region
