# Supplementary material for: HIV-1 pol Diversity among Female Bar and Hotel Workers in Northern Tanzania
Source: PLoS One. 2014 Jul 8;9(7):e102258. doi: 10.1371/journal.pone.0102258 (PMC4087014; doi:10.1371/journal.pone.0102258)
Supplement: Table S2 — HIV-1 single and multiple variants of the same subtype among female bar and hotel workers in Moshi, Kilimanjaro region, Tanzania. (DOCX) [file pone.0102258.s003.docx]

**Table S2: HIV-1 single and multiple variants of the same subtype among female bar and hotel workers in Moshi, Kilimanjaro region, Tanzania.**

| **Subject code** | ***pol* no of quasispecies** | ***env* HIV-1 subtype** | ***pol* HIV-1 subtype** | **HIV-1 viral variant (*env*)** | **HIV-1 viral variant (*pol*)** |
| --- | --- | --- | --- | --- | --- |
| 20 | 2 | A1 | A1 | multiple | N/A |
| 46 | 24 | A1 | A1 | multiple | multiple* |
| 65 | 28 | A1 | A1 | multiple | multiple* |
| 87 | 20 | A1 | C/A1 | multiple | multiple* |
| 168 | 19 | A1 | A1 | multiple | multiple* |
| 237 | 13 | A1 | A1 | multiple | multiple* |
| 245 | 22 | A1 | A1 | multiple | multiple* |
| 620 | 18 | A1 | A1 | multiple | Single |
| 740 | 22 | A1 | D | multiple | single |
| 66 | 13 | C | C | single | multiple |
| 171 | 6 | C | C | multiple | N/A |
| 201 | 13 | C | C | multiple | single |
| 291 | 32 | C | C | multiple | multiple |

N/A: not available

* Viral quasispecies with two or more phylogentic clusters in which bootstrap support values not always significant enough to assign as a multiple variant.
